# Assessment of the Efficacy of a Mobile Phone–Delivered Just-in-Time Planning Intervention to Reduce Alcohol Use in Adolescents: Randomized Controlled Crossover Trial

**DOI:** 10.2196/16937

**Published:** 2020-05-26

**Authors:** Severin Haug, Raquel Paz Castro, Urte Scholz, Tobias Kowatsch, Michael Patrick Schaub, Theda Radtke

**Affiliations:** 1 Swiss Research Institute for Public Health and Addiction University of Zurich Zurich Switzerland; 2 Applied Social and Health Psychology University of Zurich Zurich Switzerland; 3 Center for Digital Health Interventions Institute of Technology Management University of St Gallen St Gallen Switzerland; 4 Center for Digital Health Interventions Department of Technology, Management, and Economics ETH Zurich Zurich Switzerland; 5 Health, Work & Organizational Psychology School of Psychology and Psychotherapy Witten/Herdecke University Witten/Herdecke Germany

**Keywords:** alcohol, adolescents, planning intervention, just-in-time intervention, crossover trial

## Abstract

**Background:**

Interventions to reduce alcohol use typically include several elements, such as information on the risks of alcohol consumption, planning for sensible drinking, and training of protective behavioral strategies. However, the effectiveness of these individual intervention elements within comprehensive programs has not been addressed so far, but it could provide valuable insights for the development of future interventions. Just-in-time interventions provided via mobile devices are intended to help people make healthy decisions in the moment and thus could influence health behavior.

**Objective:**

The aim of this study was to test the proximal effects of a mobile phone–delivered, just-in-time planning intervention to reduce alcohol use in adolescents who reported recent binge drinking. The efficacy of this individual intervention element was tested within a comprehensive intervention program to reduce problem drinking in adolescents.

**Methods:**

The study had an AB/BA crossover design, in which participants were randomly allocated to (1) a group receiving the planning intervention (A) in period 1 and assessment only (B) in period 2 or (2) a group receiving assessment only (B) in period 1 and the planning intervention (A) in period 2. The planning intervention included a text message to choose one of two predetermined if-then plans to practice sensible drinking with friends or when going out and a prompt to visualize the chosen plan. There was a washout period of at least 1 week between period 1 and period 2.

**Results:**

Out of 633 program participants who recently binge drank, 136 (21.5%) were receptive in both periods of time and provided data on the proximal outcome, which was the number of alcoholic drinks consumed with friends or when going out. After the planning intervention, the number of alcoholic drinks consumed was approximately one standard drink lower compared with the finding without the intervention (P=.01).

**Conclusions:**

A mobile phone–delivered, just-in-time, if-then planning intervention to practice sensible drinking with friends or when going out is effective in reducing alcohol consumption among adolescents who report recent binge drinking. Based on the relatively low percentage of participants with self-reported receptivity for the planning intervention, measures to increase the population impact of similar planning interventions should be implemented and tested in future trials.

**Trial Registration:**

ISRCTN Registry ISRCTN52150713; http://www.isrctn.com/ISRCTN52150713

## Introduction

Alcohol use is a major cause of disease burden in most countries worldwide and is among the 10 leading risk factors in all Central European countries [1]. In young people, drinking is associated with multiple social and interpersonal problems, such as arguing with friends and parents, engaging in unplanned sexual activity, drinking and driving, assault, getting into trouble with the law, academic difficulties, unintended injuries, and suicidal acts [2,3]. In the long term, individuals with problematic alcohol use exhibit an elevated risk of developing chronic conditions, such as heart and liver diseases and alcohol use disorders.

Internationally recognized indicators of problem drinking are (1) average daily consumption of more than two standard drinks for men and one standard drink for women [4] and (2) binge drinking, which is defined as drinking at least five standard drinks on a single occasion for men and four drinks on a single occasion for women [5]. In particular, binge drinking prevalence rates are high in adolescence and young adulthood. In Switzerland, the binge drinking prevalence on a monthly basis is 25% in adolescents aged 15 to 19 years and 41% in young adults aged 20 to 24 years [6]. The prevalence of elevated mean daily consumption in young people is low (2% at 15–19 years of age and 8% at 20–24 years of age) relative to binge drinking, and it almost always occurs in combination with binge drinking [6].

Interventions, including personalized normative feedback and drinking reduction strategies, as major intervention elements show small short-term effects on the reduction of binge drinking prevalence in young people [2]. These intervention elements are typically included in comprehensive intervention programs, which include several elements derived from major psychological models of health behavior change, such as social norms, outcome expectations, motivation, self-efficacy, and planning interventions. Planning interventions, including *if-then plans*, are among the most recognized and frequently applied planning techniques adopted to change health behavior [7]. These strategies, also known as implementation intentions, require people to specify a critical situation and pair it with a goal-directed behavioral response (if situation *x* occurs, then I will show behavior *y*). Behavioral responses could be self-generated or prespecified, as in the intervention of this study. Laboratory research showed that both prespecified and self-generated if-then planning interventions were effective to reduce alcohol use in a representative sample of adults [8] and in alcohol-consuming adolescents [9]. Beyond laboratory research, the proximal effects of specific intervention elements (so called microinterventions like implementation intentions) might also be tested within comprehensive intervention programs. Testing the effects of these microinterventions within traditionally delivered comprehensive intervention programs allows balancing internal and external validities in a way that facilitates translation and testing of the basic theory in multicomponent intervention programs [10]. According to this approach, the effect of the intervention element could be studied within real-life conditions and assignment of participants to a control group without any intervention is not necessary. Unlike traditional methods of delivering planning interventions, mobile phones can deliver these microinterventions “just in time” for when a person is most vulnerable and receptive. These just-in-time interventions can be activated by users themselves (user triggered) through prespecified rules (server triggered, like in this study) or sensors that dynamically monitor a user’s context (context triggered) [11]. They are intended to support an individual at the time when most needed [12]. To date, published studies on the effects of just-in-time interventions are limited to physical activity and sedentary behavior, with mixed evidence for intervention effects [13].

In this study, we tested the proximal effects of a mobile phone–delivered, just-in-time, if-then planning intervention to reduce alcohol use in adolescents who reported recent binge drinking. We hypothesized that the number of alcoholic drinks consumed with friends or when going out would be lower during the just-in-time planning intervention as compared with assessment only.

## Methods

### Study Objectives and Design

The study aimed to determine the proximal effects of a just-in-time planning intervention to reduce alcohol use in adolescents who reported recent binge drinking. The study was registered at Current Controlled Trials International Standard Randomized Controlled Trials Number (ISRCTN 52150713, assigned June 2, 2017). The study protocol was approved by the ethics committee of the Philosophical Faculty at the University of Zurich, Switzerland (date of approval: April 18, 2017). The trial was executed in compliance with the Declaration of Helsinki.

Within a randomized controlled AB/BA crossover design, each participant received the planning intervention (A) and assessment only (B) in a randomized order. The trial was conducted in Switzerland, and participants were recruited between June 2017 and July 2018. Participants were recruited in vocational and upper secondary schools and participated in a comprehensive mobile phone–based intervention program to reduce problem drinking with a duration of 3 months. The inclusion criteria were (1) ownership of a mobile phone, (2) recent binge drinking, (3) alcohol consumption in the evening/night with friends or when going out, and (4) available data on preferred if-then plans. The just-in-time planning intervention was based on effective implementation intention and action planning interventions [7]. On two of their typically indicated drinking days, participants either received (A) a text message to choose one of two predetermined if-then plans to practice sensible drinking with friends or when going out and subsequently another text message prompting to visualize the chosen plan or (B) no intervention. There was a washout period of at least 1 week between A and B as well as B and A.

### Procedure and Participants of the Comprehensive Intervention Program

Vocational and upper secondary schools in the Swiss cantons of Zurich and Berne were invited to participate in the comprehensive mobile phone–based intervention program named *MobileCoach Alcohol* by prevention specialist centers. Sixteen vocational and upper secondary schools, with 108 classes in total, agreed to participate in the program and the study. Research assistants (psychology master’s degree students or graduates) invited all of the students in the participating classes to take part in an online health survey during a regular school lesson reserved for health education. Online screening was conducted using tablet computers provided by the research assistants or the students’ own smartphones. Demographic data, alcohol consumption, smoking status, and mobile phone ownership were assessed. The only inclusion criterion for participation in the comprehensive program was ownership of a mobile phone. A total of 1710 students were present in the school classes. Out of these, 1676 participated in the online screening and 1419 (83.0% of the students present in the classes) consented to participate in the *MobileCoach Alcohol* intervention program and provided their mobile phone number. Program participants were informed about data protection, the aims of the program and study, assessments, and reimbursement. Informed consent was obtained online from all program and study participants. The automated intervention program included online feedback provided immediately after the baseline assessment and individually tailored text messages provided over 3 months. The online feedback included normative feedback based on the social norms approach [14]. The text messages for binge drinking participants focused on (1) motivation to drink within low-risk limits, using individual data concerning positive outcome expectancies [15]; (2) alcohol-related problems, established using individual data on previous alcohol-related problems; (3) peak blood alcohol concentration and related risk calculated using data concerning sex, body weight, and maximum number of drinks consumed on a single occasion in the preceding month; and (4) strategies to resist alcohol when going out or when being with friends. Additionally, three text message assessments were performed during the intervention period. First, a quiz on the metabolism of alcohol, for which participants received immediate individualized feedback on their answers, and if they did not respond within 48 hours, they were sent the correct responses. Second, a contest that required participants to create a text message to motivate other participants to drink within low-risk limits. The best text message, rated weekly by an alcohol prevention specialist from the Swiss Research Institute for Public Health and Addiction, was sent anonymously to all other participants after 48 hours. Third, an assessment of binge drinking within the preceding week, which included immediate individualized feedback. The text messages typically contained 150 to 300 characters and were delivered via SMS text messaging. Several text messages also included web links to thematically appropriate video clips, pictures, and websites. Except for the text messages providing the planning intervention, which were typically sent on Fridays and Saturdays at 5 pm (mentioned below), messages were typically sent on Tuesdays at 6 pm.

### Procedure and Participants of the Planning Intervention Study

**Figure 1:**
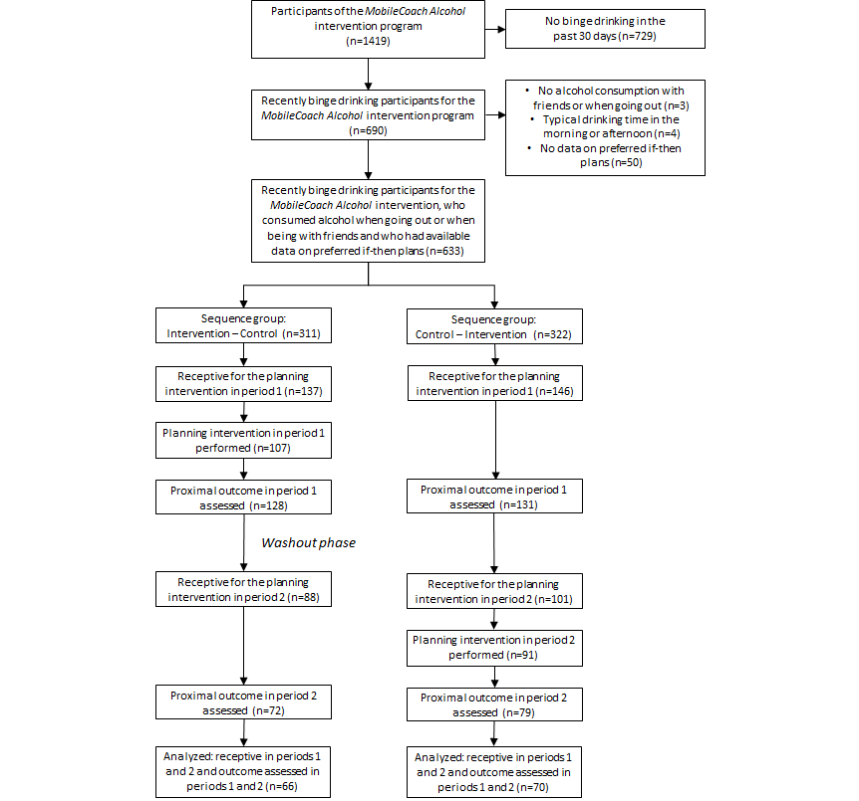
Flow of study participants.

The participants for this study to test the proximal effects of the mobile phone–delivered, just-in-time, alcohol planning intervention were selected automatically by a computer algorithm within the *MobileCoach Alcohol* intervention program (Figure 1).

The computer algorithm selected recent binge drinking adolescents with alcohol consumption in the evening or night when going out or when being with friends and with available data on preferred if-then plans. A total of 633 (44.6%) of the 1419 *MobileCoach Alcohol* intervention program participants fulfilled the inclusion criteria and were randomly allocated to (1) a group receiving the planning intervention (A) in period 1 and assessment only (B) in period 2 or (2) a group receiving assessment only (B) in period 1 and the planning intervention (A) in period 2. The randomization sequence with a 1:1 ratio was created using computerized random numbers.

**Figure 2:**
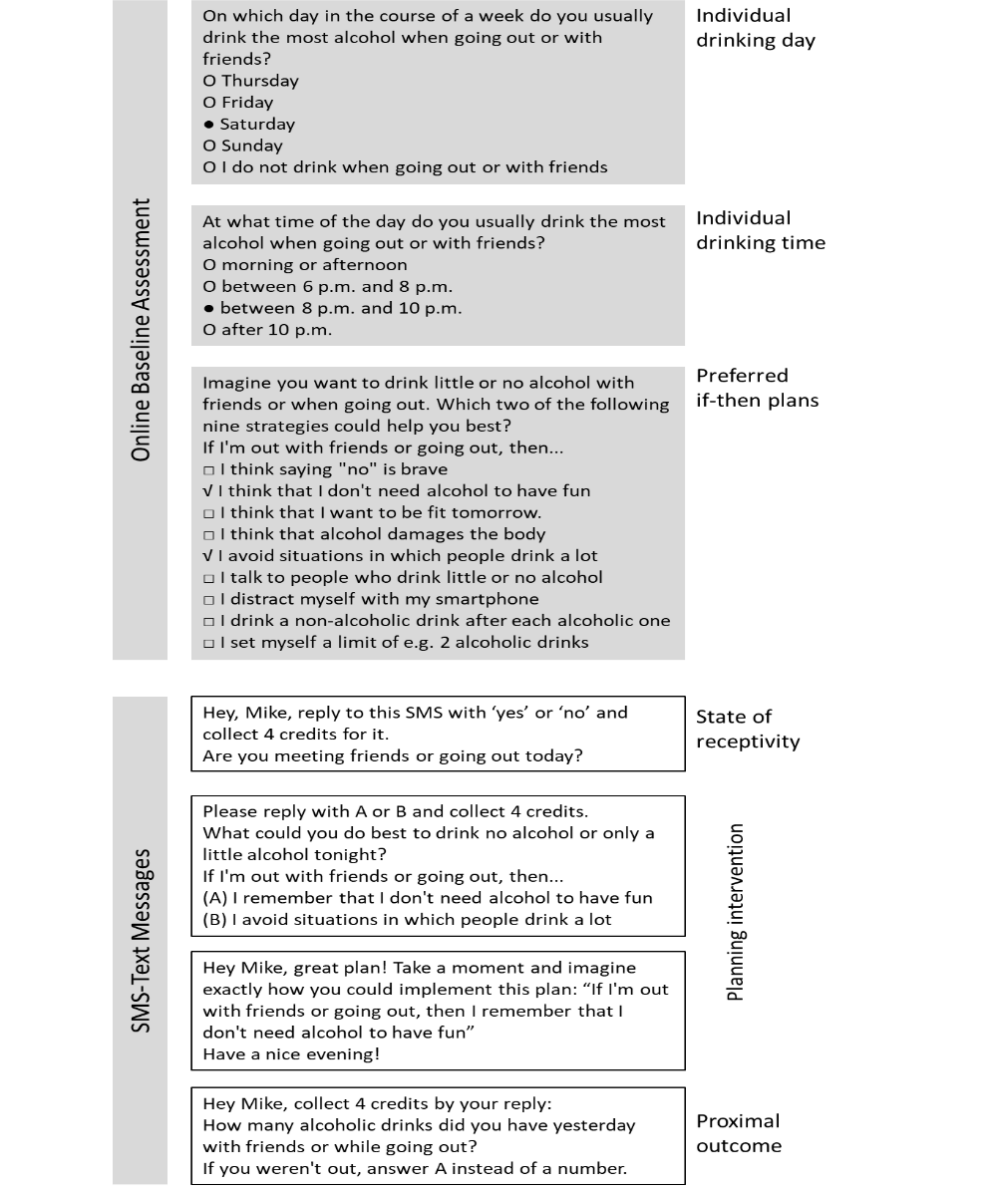
Assessments and planning intervention from participants' perspective.

The assessment questions and the text messages of the planning intervention are depicted in Figure 2.

The data necessary for the provision of the just-in-time planning intervention were assessed within the baseline assessment. This assessment was also performed during the school lesson immediately after the online screening and informed consent procedure for the comprehensive intervention program (mentioned above). It included (1) selection of the typical drinking day in the course of the week when going out or when being with friends, (2) selection of the typical drinking time when going out or when being with friends, and (3) selection of two out of nine favorite if-then plans providing strategies to drink little or no alcohol with friends or when going out. One reason to select only two out of nine if-then plans was that these plans were also presented within SMS text messages, which are restricted in their length and number of characters. Another reason was not to overstrain the participant within this situation. Regarding the typical drinking days, we did not include Monday, Tuesday, and Wednesday, as previous studies [16,17] on this program showed that practically nobody chose these days. Furthermore, we wanted to prevent temporal overlap of the just-in-time intervention elements (presented Thursday to Sunday) and the other intervention elements of the program, which were typically sent on Tuesdays. The text messaging–based part included (1) assessment of the state of receptivity on the individually indicated drinking day at 5 pm, (2) the actual planning intervention comprising a text message to choose one of two predetermined if-then plans to practice sensible drinking with friends or when going out and another text message prompting to visualize the chosen plan, and (3) assessment of the proximal outcome (the number of alcoholic drinks consumed on the previous day with friends or when going out). The assessment-only condition solely included (1) and (3). The planning intervention was designed considering the latest recommendations for research and practice on planning and implementation intentions in health contexts [7].

As the entire intervention program had a total duration of 12 weeks and we considered a washout period of 1 week as appropriate between period 1 and 2, assessments of the state of receptivity and potential provision of the subsequent planning intervention were possible six times during the intervention (in weeks 1, 3, 5, 7, 9, and 11). In order to obtain a maximum sample size for this crossover trial, the state of receptivity was assessed in as many weeks as possible until a participant was receptive twice, that is, there were up to six chances for the period 1 assessment, and the remaining assessments after responding to the period 1 assessment were for period 2. After being receptive twice, the participants no longer received this state of the receptivity assessment. The time interval for responding to the state of the receptivity assessment was 6 hours. Participants who did not respond within this time period and those who indicated that they did not meet with friends or go out did not receive the subsequent messages of the planning intervention and the outcome assessment.

### Sample Size Calculation

The estimation of the effect size was based on the results of a controlled study testing the effectiveness of implementation intentions to reduce alcohol use in a sample of the general population [8], using an online power calculator for crossover studies provided by the Biostatistics Center of Massachusetts General Hospital [18]. Within this study [8], the participants randomized to the experimenter-provided implementation intention condition were presented with a choice of three implementation intentions, from which they chose the one they thought would work best for them and wrote it down. Participants with high alcohol intake under this condition reduced their alcohol intake from baseline to a 1-month follow-up by 1.3 standard drinks per day, whereas this reduction was 0.1 drinks in the passive control group. Using this minimal detectable difference in means of 1.2, a standard deviation within participants of 3.0, a power of 80%, and a noncentral *t* function (α=5%, one-sided), the study required a total of 79 participants.

### Assessments and Outcomes

The online baseline assessment included the following variables: sex, age, immigration background, tobacco smoking, the Alcohol Use Disorders Identification Test (AUDIT) [19], and binge drinking. We assessed the countries of birth for students’ parents to identify a potential immigrant background. Based on this information, participants were assigned to one of the following categories: (1) neither parent born outside Switzerland, (2) one parent born outside Switzerland, and (3) both parents born outside Switzerland. In the analysis, we combined one- and two-sided immigrant backgrounds into a single category and compared it with a nonimmigrant background.

Tobacco smoking was assessed using the following question: “Do you currently smoke cigarettes or have you smoked in the past?” The response options were as follows: (1) I smoke cigarettes daily; (2) I smoke cigarettes occasionally but not daily; (3) I smoked cigarettes in the past, but I do not smoke anymore; and (4) I have never smoked cigarettes or have smoked less than 100 cigarettes throughout my life. In the analysis, we combined categories (1) and (2) as smokers and categories (3) and (4) as nonsmokers. Alcohol use was assessed through the consumption items of the AUDIT (AUDIT-C) [19]. The AUDIT-C assesses drinking quantity, drinking frequency, and binge drinking frequency, and it has a potential range from 0 to 12, with higher values representing higher alcohol use. Binge drinking prevalence in the preceding 30 days was assessed by asking participants to report the number of standard drinks consumed on the heaviest drinking occasion in the preceding 30 days. Examples of standard drinks containing 12 to 14 g of ethanol were provided for beer, wine, spirits, alcopops, and cocktails, along with conversion values (eg, three 0.5 L cans of beer equals six standard drinks). Binge drinking was defined as drinking at least five drinks on a single occasion in males and four drinks on a single occasion in females [5].

The primary outcome of this planning intervention study was the number of alcoholic drinks with friends or when going out on the day of the intervention. This proximal outcome was assessed 24 hours after assessment of the state of receptivity, that is, at 5 pm on the day following the individual indicated drinking day. The secondary outcome of this planning intervention study was binge drinking, which was defined as drinking at least five drinks in males and four drinks in females with friends or when going out on the individual indicated drinking day.

### Data Analysis

We initially examined the data of the primary outcome, which was based on the self-reported number of alcoholic drinks entered as free text. Based on a visual inspection of the distributions and the recommendations of Osborne and Overbay [20], outliers were identified at more than 3 standard deviations above the mean and adjusted to 3 standard deviations above the mean.

We used chi-square tests for categorical variables and *t* tests for continuous variables to evaluate baseline differences between the analyzed sample and the sample of participants not responding or receptive in both periods of time in order to examine the representativeness of the sample.

Intervention effects of the planning intervention were tested following the recommendations of Wellek and Blettner [21] on proper data analysis in clinical trials with a crossover design. These recommendations include the following: (1) participants are assigned randomly to the two sequence groups AB and BA; (2) the crucial variable for analysis is the within-subject difference in outcome between the two study periods, tested by a valid test for independent samples comparing the values of this variable between the sequence groups; and (3) an assumption that the washout phase is long enough to rule out a carryover effect, which should be checked by another test for independent samples.

Following the latter recommendation and to rule out that the treatment effects were confounded by time effects or a carryover effect, as the washout phase was not long enough, we calculated the sum of the number of drinks consumed in the two periods for each subject and compared it across the two sequence groups by a *t* test for independent samples.

To test for the effects of the alcohol planning intervention, we used a *t* test for independent samples for the primary outcome (number of alcoholic drinks on the previous day with friends or when going out) and a chi-square test for the binary secondary outcome (binge drinking on the previous day with friends or when going out). All outcome analyses were based on a complete-case dataset, which included participants who were receptive in periods 1 and 2 and had outcomes assessed in both time periods. Results with a type I error rate involving a *P* value <.05 in two-sided tests were considered statistically significant, with the exception of the proximal outcome that was tested one-sided, as the study hypotheses and power calculations were also based on one-sided tests. Analyses were performed using SPSS, version 22 (IBM Corp, Armonk, New York, USA).

## Results

### Study Participation

Figure 1 depicts the participants’ progression through the trial. A total of 633 binge drinking participants for the *MobileCoach Alcohol* intervention, who consumed alcohol in the evening or night when going out or when being with friends and who had available data on preferred if-then plans, were randomly allocated to the sequence group AB (n=311) or the sequence group BA (n=322). Out of these 633 participants, 136 (21.5%) were receptive twice and provided data on the proximal outcome at both time points. Our analyses on the effectiveness of the just-in-time alcohol planning intervention were based on these 136 participants.

This analyzed group did not differ from participants who could not be assessed (n=497) with respect to the following baseline characteristics: age, immigration background, tobacco smoking status, AUDIT-C, typical drinking day, and typical drinking time on the indicated drinking day. However, the nonreceptive participants or nonresponders were more likely male (51.5% vs 40.8%, χ^2^_1_=4.9; *P*=.03) and in upper secondary than vocational schools (45.5% vs 32.4%; χ^2^_1_=7.5; *P*=.006).

Characteristics of the analyzed study sample (n=136) and the two sequence groups (AB/BA) are shown in Table 1.

**Table 1:** Baseline characteristics of the study sample.

Variable		Sequence: intervention–control (AB) (n=66)	Sequence: control–intervention (BA) (n=70)	Total (n=136)
**Sex, n (%)**				
	Male	35 (53.0%)	35 (50.0%)	70 (51.5%)
	Female	31 (47.0%)	35 (50.0%)	66 (48.5%)
Age, mean (SD)		16.9 (1.0)	17.2 (1.3)	17.1 (1.1)
**Immigration background, n (%)**				
	No immigration background	30 (45.5%)	42 (60.0%)	72 (52.9%)
	One or both parents born outside Switzerland	36 (54.5%)	28 (40.0%)	64 (47.1%)
**Type of school, n (%)**				
	Upper secondary school	20 (30.3%)	24 (34.3%)	44 (32.4%)
	Vocational school	46 (69.7%)	46 (65.7%)	92 (67.6%)
**Tobacco smoking status, n (%)**				
	Daily or occasional cigarette smoking	31 (47.0%)	34 (48.6%)	65 (47.8%)
	Nonsmoking	35 (53.0%)	36 (51.4%)	71 (52.2%)
AUDIT-C^a^, mean (SD)		6.2 (1.6)	6.7 (1.7)	6.4 (1.7)
**Typical drinking day, n (%)**				
	Thursday	1 (1.5%)	0 (0%)	1 (0.7%)
	Friday	28 (42.4%)	27 (38.6%)	55 (40.4%)
	Saturday	37 (56.1%)	43 (61.4%)	80 (58.8%)
	Sunday	0 (0%)	0 (0%)	0 (0%)
**Typical drinking time on the indicated typical drinking day, n (%)**				
	Morning or afternoon	0 (0%)	0 (0%)	0 (0%)
	Between 6 pm and 8 pm	4 (6.1%)	5 (7.1%)	9 (6.6%)
	Between 8 pm and 10 pm	32 (48.5%)	40 (57.1%)	72 (52.9%)
	After 10 pm	30 (45.5%)	25 (35.7%)	55 (40.4%)

^a^AUDIT-C: consumption items of the Alcohol Use Disorders Identification Test

### Test for Period Effects

To rule out that the treatment effects were confounded by time effects or carryover effects, we calculated the sum of the number of drinks consumed in the two periods for each subject and compared it across the two sequence groups by a *t* test for independent samples. This test did not suggest that any time or period effects were present (*t*=−1.19, *P*=.24).

### Effects of the Alcohol Planning Intervention

As shown in Table 2, in the AB and BA sequence groups, the mean numbers of alcoholic drinks consumed on the previous day with friends or when going out were 2.79 and 3.43, respectively, after receiving the planning intervention and 3.68 and 4.21, respectively, without the intervention. The within-subject differences of 0.89 and −0.79 between the sequence groups were statistically significant (*t*=2.31, *P*=.01), showing that the planning intervention is effective to reduce the mean alcohol use on typical drinking days in young people by about one standard drink.

Concerning the secondary outcome (binge drinking on the previous day with friends or when going out), in the AB and BA sequence groups, the prevalences were 30% (20/66) and 36% (25/70), respectively, after receiving the planning intervention and 35% (23/66) and 36% (25/70), respectively, without the intervention. A chi-square test comparing the number of participants who did not change between period 1 and period 2, who reported binge drinking in period 2 but not in period 1, and who reported binge drinking in period 1 but not in period 2, showed no statistically significant difference between the two sequence groups (*χ^2^*_2_=1.34, *P*=.25).

**Table 2:** Effects of the alcohol planning intervention.

Variable	Sequence: intervention–control (AB) (n=66)			Sequence: control–intervention (BA) (n=70)			Test value	*P* value
	Period 1 (A)	Period 2 (B)	Within-subject difference (period 2−period 1)	Period 1 (B)	Period 2 (A)	Within-subject difference (period 2−period 1)		
Number of alcoholic drinks on the previous day with friends or when going out, mean (SD)	2.79 (3.09)	3.68 (3.62)	0.89 (3.71)	4.21 (3.67)	3.43 (3.85)	−0.79 (4.69)	2.31^a^	.01
Binge drinking on the previous day with friends or when going out, n (%)	20 (30%)	23 (35%)	3 (5%)	25 (36%)	25 (36%)	0 (0.0%)	1.34^b^	.25

^a^*t* test for independent samples for the comparison of the within-subject difference between the condition sequences.

^b^Chi-square test for the comparison of binge drinking change in period 2 compared with period 1 between the condition sequences.

## Discussion

This study aimed to test the proximal effects of a just-in-time planning intervention for reducing alcohol use in adolescents who reported recent binge drinking. To the best of our knowledge, this is the first study to test the effects of a just-in-time intervention for reducing alcohol consumption. The study revealed the following three main results, which are discussed below: (1) It is feasible to test the proximal effects of single intervention elements like implementation intentions within a comprehensive multicomponent intervention program if the program is delivered via a mobile phone and has a minimum duration; (2) An if-then alcohol planning intervention is effective to reduce the mean alcohol use on typical drinking days in young people by about one standard drink; (3) A large proportion of adolescents is not receptive to the just-in-time planning intervention, when the state of receptivity is assessed via text messaging and a reply within a limited response time is required for triggering the intervention.

This study underlines that the proximal effects of specific intervention elements like just-in-time implementation intentions could be tested in a randomized controlled crossover design within a comprehensive intervention program, if some requirements are met as follows: (1) The intervention program is provided automatically (eg, via a mobile phone that allows server-triggered just-in-time interventions); (2) The duration of the program is long enough to assess receptivity for the intervention several times in order to have at least two time points for comparison; and (3) The intervention element is presented after a long enough time (wash-out period) from the other elements or contents of the program in order to reduce confounding. Another requirement, which was hardly met in previous trials on just-in-time interventions, is an adequate sample size to have enough statistical power [13]. Currently published trials in the areas of physical activity and sedentary behavior typically have much smaller sample sizes than this trial, although the state of receptivity and proximal outcomes were often assessed automatically and unobtrusively via smartphone sensors or activity trackers [22].

The result that a single if-then planning intervention is effective to reduce alcohol consumption is in line with previous findings derived from laboratory research [8,9] and extends the insights to that effect that the planning intervention could also be provided digitally without any personal contact, as well as just-in-time on the individually indicated drinking day. Owing to different follow-up intervals, the achieved reduction in alcohol use could not directly be compared with other interventions. However, to enable a rough estimation, a traditional if-then planning intervention in adolescents resulted in an average reduction of 2.5 g of alcohol per day (one-fifth of a standard drink) at a 2-month follow-up [9]. An earlier version of the digital *MobileCoach Alcohol* intervention program resulted in an average reduction of 0.4 standard drinks per day at a 6-month follow-up [17].

Although the just-in-time delivery of the alcohol planning intervention might be partly responsible for its effectiveness, it remains unclear whether the population impact (number of participants reached multiplied by effectiveness [23]) of the provided planning intervention might have been greater if selection of the preferred if-then plan would have taken place within a longer timeframe before the drinking day (eg, the whole day before) or an initially preferred if-then plan would have been sent irrespective of the state of receptivity. Both options may be able to increase the impact of this planning intervention as compared with the procedure implemented in this trial, where an active reply on receptivity via text messaging within a limited response time was required. However, direct comparisons of different delivery options or qualitative participant interviews are necessary for a better understanding of the limited reach. Additional analyses of the replies to the receptivity question “Are you meeting friends or going out today?” showed that a substantial part of the lack of receptivity was due to the denial of this question; in weeks 1 and 3 when all study participants received this question, 231 out of 492 (47.0%) denied meeting friends or going out that day. These data suggest that half of adolescents are not receptive because they are not at risk for binge drinking on a predetermined day. Correspondingly, measures to update or adapt the drinking day during program delivery might be promising to increase receptivity.

Several limitations of this study should be mentioned. First, the number of alcoholic drinks consumed was self-reported, and in contrast to the baseline assessment, no examples of standard drinks were provided within the text messaging–based assessment of the proximal outcome. Second, although the crossover design applied has several advantages (eg, it avoids problems of comparability of the study and control groups because each participant is his/her own control and the required sample size is low), carryover effects might have confounded part of the intervention effects, although the respective finding was not relevant. Third, the sample analyzed within this study systematically differed from all participants randomized, with respect to sex and type of school, which limits the external validity of the results. Fourth, we solely used baseline data for tailoring the intervention time. The possibility of updating or adapting the intervention time periodically might increase intervention effectiveness.

In conclusion, this study shows that just-in-time interventions could be tested and implemented in the area of addiction and that digitally provided alcohol planning interventions could reduce alcohol use in adolescents who report recent binge drinking. Future studies should focus on increasing the reach and outcome of just-in-time alcohol planning interventions by testing other delivery formats or by sensor-triggered intervention delivery, which, for example, dynamically monitors a participant’s context and provides support when high-risk environments, such as areas with many nightlife locations and social situations, are sensed [24,25].

## Appendix

Multimedia Appendix 1 CONSORT-eHEALTH checklist (V 1.6.1).

## Abbreviations

AUDIT: Alcohol Use Disorders Identification Test
AUDIT-C: consumption items of the Alcohol Use Disorders Identification Test
